# Mortality from heart failure, acute myocardial infarction and other ischaemic heart disease in England and Oxford: a trend study of multiple-cause-coded death certification

**DOI:** 10.1136/jech-2015-205689

**Published:** 2015-07-01

**Authors:** Kazem Rahimi, Marie Duncan, Alex Pitcher, Connor A Emdin, Michael J Goldacre

**Affiliations:** 1 George Institute for Global Health, Nuffield Department of Population Health, University of Oxford, Oxford, UK; 2 Division of Cardiovascular Medicine, Radcliffe Department of Medicine, University of Oxford, Oxford, UK; 3 Unit of Health-Care Epidemiology, Nuffield Department of Population Health, University of Oxford, Oxford, UK

**Keywords:** Cardiovascular disease, CHD/CORONORARY HEART, EPIDEMIOLOGY

## Abstract

**Background:**

Age-standardised death rates from acute myocardial infarction (AMI) and ischaemic heart disease (IHD) have been declining in most developed countries. However, the magnitude of such reductions and how they impact on death from heart failure are less certain. We sought to assess and compare temporal trends in mortality from heart failure, AMI and non-AMI IHD over a 30-year period in England.

**Methods:**

We analysed death registration data for multiple-cause-coded mortality for all deaths in people aged 35 years and over in England from 1995 to 2010, population 52 million, and in a regional population (Oxford region) from 1981 to 2010, population 2.5 million, for which data on all causes of death were available.

**Results:**

Considering all ages and both sexes combined, during the 30-year observation period, age-standardised and sex-standardised mortality rates based on all certified causes of death declined by 60% for heart failure, 80% for AMI and 46% for non-AMI IHD. These longer term trends observed in the Oxford region were consistent with those for the whole of England from 1995 to 2010, with no evidence of a plateau in recent years. Although proportional reductions in rates differed by age and sex, even in those aged 85 years or more, there were substantial reductions in mortality rates in the all-England data set (50%, 66% and 20% for heart failure, AMI and non-AMI IHD, respectively).

**Conclusions:**

This study shows large and sustained reductions in age-specific and sex-specific and standardised death rates from heart failure, as well as from AMI and non-AMI IHD, over a 30-year period in England.

## Introduction

Heart failure is the end stage of several cardiovascular and non-cardiovascular disease processes. The Global Burden of Disease study estimated that in 2010, 37% of all cases of symptomatic heart failure worldwide were causally attributable to myocardial infarction alone,^1^ and a collaborative review of heart failure studies in low-income and middle-income countries came to a similar conclusion and reported ischaemic heart disease (IHD) to be responsible for 35% of heart failure cases.^2^ However, despite the strong overlap between IHD and evidence suggesting substantial declines in age-adjusted prevalence of fatal and non-fatal myocardial infarction in developed countries,^3^ reports on the burden of heart failure as a consequence of those conditions have been less consistent.

The 2013 American College of Cardiology Foundation/American Heart Association guideline for management of heart failure, for example, suggests a continuous increase in the prevalence of heart failure in the USA,^4^ based on a study from an elderly Medicare population in the USA that showed a rise in prevalence from 90 to 121 cases per 1000 beneficiaries from 1994 to 2003.^5^ A similar rise in age-adjusted prevalence was reported in another study based on the Olmsted County cohort in Minnesota from 1979 to 2000.^6^ However, a more comprehensive analysis of the Medicare data showed a 30% relative decline in heart failure hospitalisations just within a decade.^7^ Analyses of death certificates in several high-income countries have also shown declines in age-adjusted heart failure death rates,^8^ ^9^ although some recent national analyses have suggested that mortality rates for heart failure reached their nadir in 2005 and have even started to increase again.^10^

Mortality statistics are an important source of information on disease trends at the population level. However, estimation of the burden of heart failure based on death certificates can be problematic because heart failure is the phenotypic presentation of several other diseases and is not a disease, in and of itself. Consequently, analysis of causes of death is likely to underestimate its true prevalence.^11^ Further, coding rules for mortality statistics discourage the recording of heart failure as the underlying cause of death when other diseases are present and are considered to have contributed to death. In the majority of death certificates that include heart failure, heart failure is not coded as the underlying cause. Accordingly, studies of heart failure deaths require access to data that include all certified causes of death (not just the underlying cause).

The Oxford region of England has the longest run of multiple cause-coded mortality data in England, dating back more than 30 years. Using this data set, we sought to quantify the trend in death with (not only from) heart failure over a period longer than has hitherto been possible and to relate this trend to that of deaths with IHD.

## Methods

Nationally in England, from 1993 onwards, handwritten causes of death in England have been digitised by the local Registrar of Births, Deaths and Marriages, and sent electronically to the Office for National Statistics (ONS) for processing. Specific text terms from the death certificate are converted by the ONS to the International Classification of Disease (ICD) codes. Selection and modification rules are then assigned to determine the underlying cause of death. The recording of all causes of death on each certificate started earlier (in 1979) in the former Oxford National Health Service (NHS) region of England as part of the Oxford Record Linkage Study (ORLS).^12^ The ORLS resource, while restricted to a particular region of England, allows comprehensive analysis of mortality data over an extended period. Our analyses used the mortality data set of the former Oxford NHS region over a 30-year period (1981–2010 inclusive), as held in the ORLS data set, covering a population of approximately 2.5 million. In addition, to set the regional data in a national context and to corroborate recent findings, we used the English national death registration data over a 16-year period (1995–2010 inclusive). These data were provided by the ONS and cover a population of about 52 million. The data sets available to us included all deaths registered up to the end of 2011. There is a time lag between occurrence of death and its registration. Accordingly, although we included registrations to the end of 2011 in the analysis, we only show deaths that occurred up to 2010 in order to provide as complete an enumeration as possible by calendar year of occurrence. Data for Oxford and England are comparable: both are provided annually as anonymised individual person-level data by the ONS.

We searched the regional and national death registration records for all deaths coded for the relevant ICD codes. The ninth revision of the ICD (ICD-9) codes cover the period 1981–2000. The ICD-10 codes were introduced in 2001, and cover the period 2001 onwards. The relevant codes selected were as follows: heart failure, ICD-9 code 428, ICD-10 code I50; acute myocardial infarction (AMI), ICD-9 code 410, ICD-10 code I21; IHD (excepting AMI), ICD-9 codes 411–414, ICD-10 codes I20 and I22–I25.

The principal analyses for each condition were performed using multiple-cause-coded mortality data. This type of analysis takes account of all conditions mentioned on the death certificate, irrespective of whether they are listed as the underlying cause, or as a contributing cause. Such certified causes—the underlying cause and other contributing causes—are conventionally called ‘mentions’, and analyses of this type are said to be ‘based on mentions’. We have adopted this terminology in this paper.

In the calculation of mortality rates, the numerators were the numbers of deaths in each age–sex group in each calendar year; and the denominators were all people in the relevant resident population in the corresponding age–sex groups. Separate analyses were performed by sex and by age at death, in 5-year age groups. Annual age-standardised mortality rates were calculated by applying the age-specific death rates for each disease, in 5-year age groups, in each population in each year to the European standard population. The ratios of rates as well as the absolute number of deaths for 2008–2010 were compared with those in 1981–1983 by simple division of the mortality rates (by sex, within each age group) and results were expressed as percentage reductions. This analysis is not an analysis of continuous trends, but a comparison of discrete rates and absolute deaths in 1981–1983 to 2008–2010.

## Results

**Figure 1:**
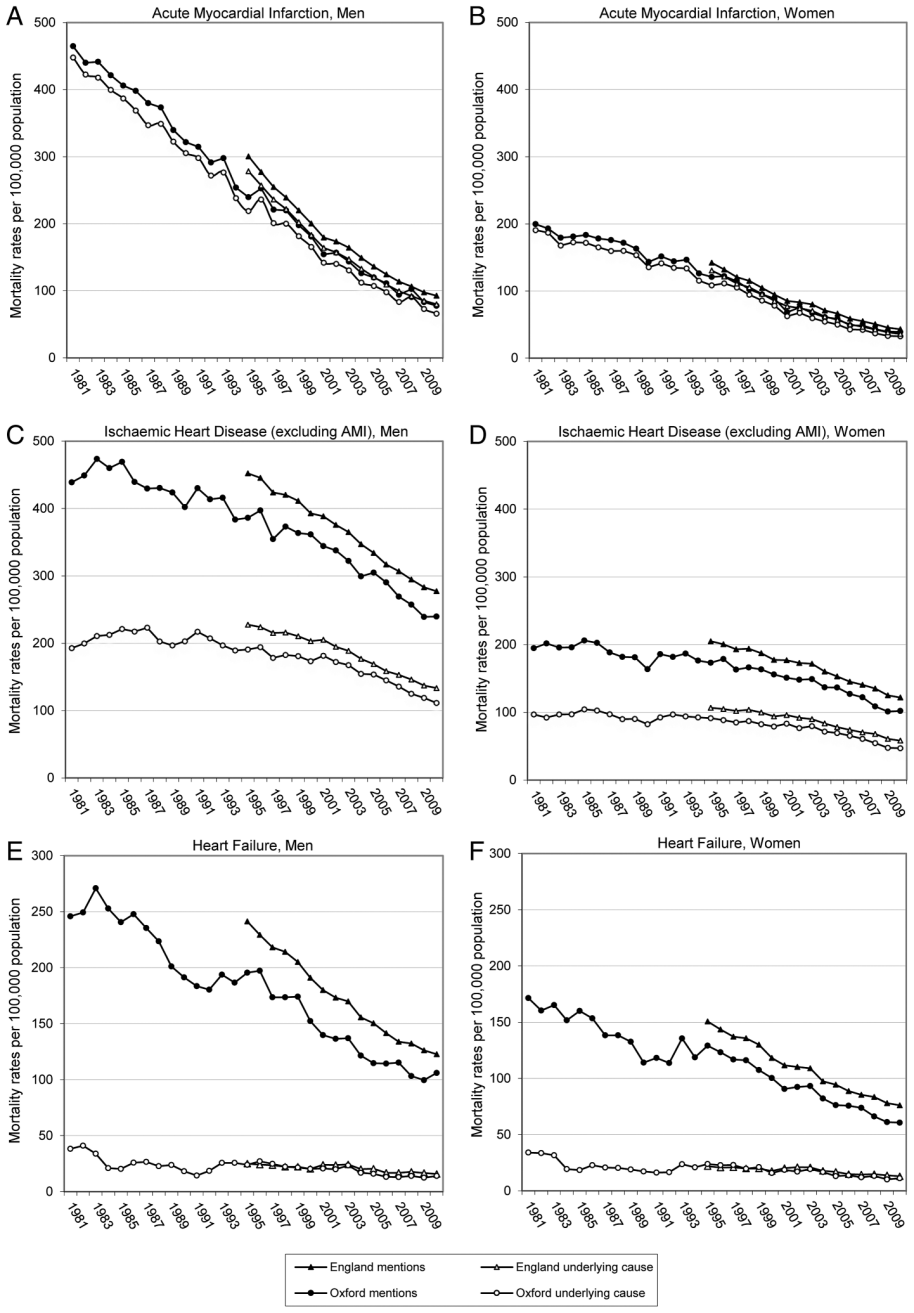
Age-standardised mortality rates for acute myocardial infarction (AMI), other ischaemic heart disease and heart failure from 1995 to 2010 for the population aged 35 years and over.

Figure 1 shows age-standardised mortality rates for heart failure, AMI and IHD (excluding AMI) in the Oxford region and in England for populations aged 35 years and over. At points where they can be compared directly, the mortality trends for both populations show the same pattern, although mortality rates tend to be lower in the Oxford region (likely a reflection of the relative affluence of the Oxford region compared with England as a whole).

### Thirty year trends in heart failure mortality

**Figure 2:**
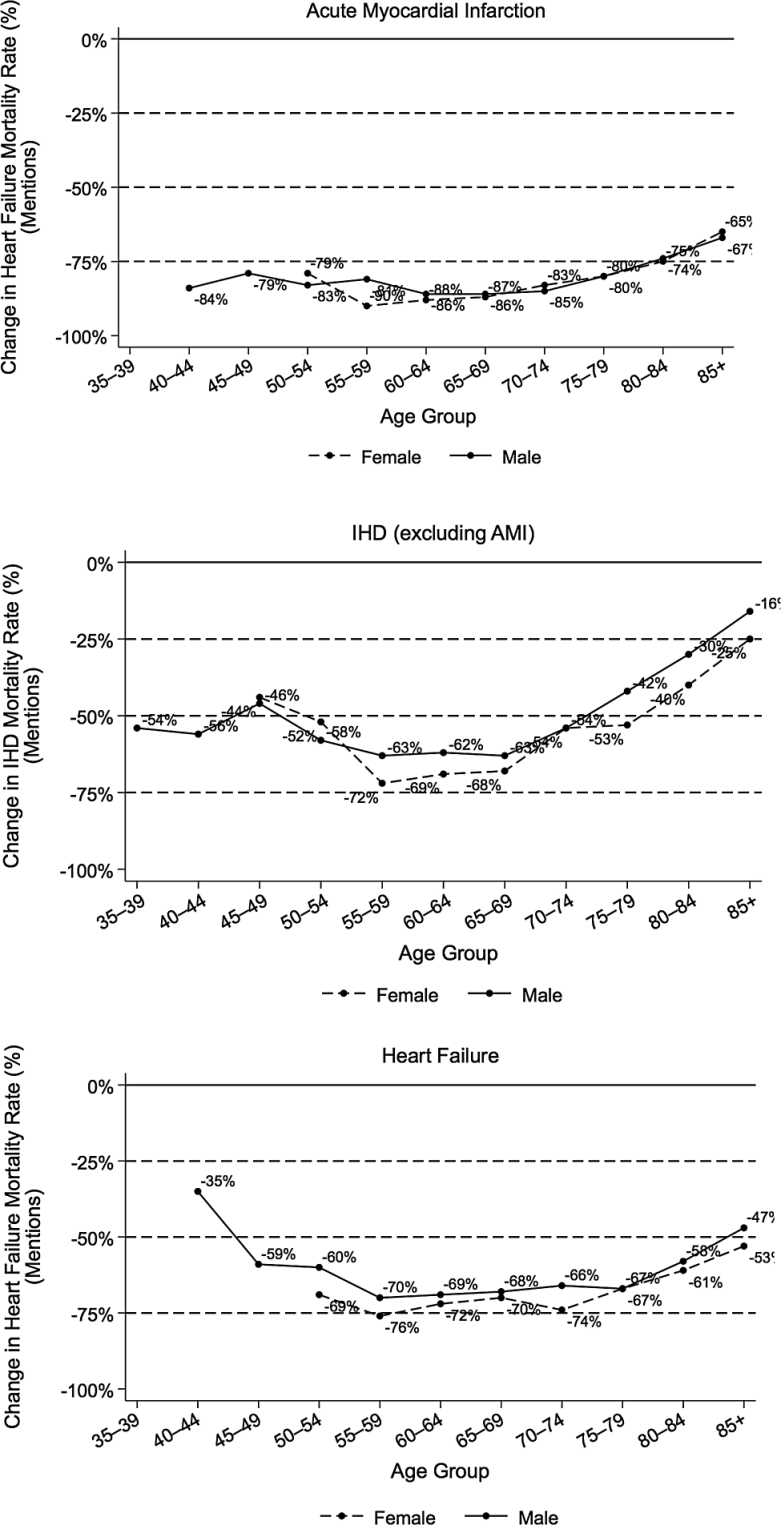
Relative change in mortality for acute myocardial infarction, ischaemic heart disease and heart failure from 1981 to 2010, stratified by age group and sex.

Large declines in mortality rates for heart failure were seen over the 30-year study period in the Oxford region, and were confirmed over the latter half of this period in the larger English national data set (figure 1 and table 1). Overall, between the first and final 3 years of the 30-year study period, mortality rates (based on all mentions of heart failure on the death certificate) decreased from 130.6 to 51.8 per 100 000 population. Across all age groups and both sexes, the age-standardised mortality rate in 2008–2010, based on all mentions of heart failure on the death certificate, was only 40% of that in 1981–1983. Every age–sex group showed a substantial decline in mortality rates across the three decades, with the lowest (though still substantial) decline in the youngest and oldest age groups (figure 2). The declines in mortality were steepest in middle-aged men and women. For example, mortality rates in women aged 65–69 years in 2008–2010 were just one-quarter of those in 1981–1983 (figure 2). These age-specific reductions show that the overall all-ages declines were not merely a reflection of the ageing population and a shift to older age. Considering all age groups and both sexes, the total number of deaths with a mention of heart failure also decreased by 27% within the 30-year study period, despite an ageing population (table 1).

**Table 1:** Heart failure in the Oxford region 1981–1983 and 2008–2010

	Number of deaths		Rates per 100 000		Ratio* of rate† in 2008–2010/ 1981–1983
Age group, years	1981–1983	2008–2010	1981–1983	2008–2010	
Men					
35–39	13	9	4.9	2.8	–
40–44	21	22	9.7	6.3	0.65
45–49	48	32	24.0	9.9	0.41
50–54	99	58	51.9	20.7	0.40
55–59	182	76	100.3	30.4	0.30
60–64	277	132	172.6	53.1	0.31
65–69	400	177	305.1	99.1	0.32
70–74	593	265	545.5	183.5	0.34
75–79	798	417	1138.4	377.4	0.33
80–84	637	597	1912.9	796.0	0.42
85+	587	1083	3623.5	1927.1	0.53
35 and over	3655	2868	255.4	103.0	0.40
Women					
35–39	5	5	1.9	1.5	–
40–44	13	4	6.3	1.1	–
45–49	11	13	5.7	4.0	–
50–54	44	20	23.6	7.2	0.31
55–59	63	21	34.5	8.2	0.24
60–64	126	53	73.6	20.7	0.28
65–69	217	82	143.1	43.6	0.30
70–74	477	141	342.4	89.9	0.26
75–79	727	291	668.8	219.3	0.33
80–84	938	549	1326.7	519.4	0.39
85+	1651	1745	3181.1	1480.1	0.47
35 and over	4272	2924	165.7	62.6	0.38
Total	7927	5792	202.6	80.3	0.40

Number of mentions on death certificates, age-specific mortality rates per 100 000 population, and the age-standardised mortality rate in 2008–2010 expressed as a ratio to that in 1981–1983.

*Ratios compare periods covered by different versions of International Classification of Disease codes and governed by different rules for the selection of underlying cause of death.

†Rates given where heart failure as a cause in 2008–2010 is ≥15.

### Thirty year trends in AMI mortality

Spectacular declines in mortality rates were observed for AMI over the 30-year study period (figure 1 and table 2). The direction and magnitude of this trend in the latter half of this period was confirmed in the national data set (figure 1). Every age–sex group showed a substantial decline in mortality rates across the three decades, with the greatest declines seen in the middle-age groups (figure 2). For example, mortality rates in women aged 55–59 years in 2008–2010 were just one-tenth of those in 1981–1983. Overall, combining all ages and both sexes, the age-standardised mortality rate based on all mentions of AMI on the death certificate was in 2008–2010 only 20% of that in 1981–1983.

**Table 2:** Acute myocardial infarction in the Oxford region 1981–1983 and 2008–2010

	Number of deaths		Rates per 100 000		Ratio* of rate† in 2008–2010/ 1981–1983
Age group, years	1981–1983	2008–2010	1981–1983	2008–2010	
Men					
35–39	45	6	16.8	1.8	–
40–44	80	21	37.0	6.0	0.16
45–49	183	63	91.4	19.4	0.21
50–54	342	88	179.4	31.4	0.17
55–59	562	146	309.8	58.5	0.19
60–64	839	178	522.7	71.6	0.14
65–69	1136	209	866.5	117.0	0.14
70–74	1309	267	1204.2	184.9	0.15
75–79	1145	352	1633.4	318.6	0.20
80–84	745	428	2237.2	570.7	0.26
85+	504	580	3111.1	1032.0	0.33
35 and over	6890	2338	448.8	87.6	0.20
Women					
35–39	4	3	1.5	0.9	–
40–44	18	4	8.7	1.1	–
45–49	23	10	12.0	3.0	–
50–54	61	19	32.7	6.8	0.21
55–59	136	20	74.5	7.8	0.10
60–64	291	53	170.0	20.7	0.12
65–69	461	77	304.1	41.0	0.13
70–74	725	139	520.5	88.6	0.17
75–79	1001	243	920.9	183.1	0.20
80–84	920	348	1301.3	329.2	0.25
85+	998	785	1922.9	665.8	0.35
35 and over	4638	1701	190.4	40.5	0.21
Total	11 528	4039	302.5	61.8	0.20

Number of mentions on death certificates, age-specific mortality rates per 100 000 population, and the age-standardised mortality rate in 2008–2010 expressed as a ratio to that in 1981–1983.

*Ratios compare periods covered by different versions **of** International Classification of Disease codes and governed by different rules for the selection of underlying cause of death.

†Rates given where deaths with acute myocardial infarction as a cause in 2008–2010 is ≥10.

### Thirty year trends in IHD mortality

IHD (excluding myocardial infarction) was mentioned on the death certificate as a cause of death with diminishing frequency over the 30-year study period (figure 1 and table 3). While substantially, these declines were not as dramatic as those for AMI and heart failure. The rate of decline was similar for both sexes and while a decline was observed in every age group, IHD mortality declined more rapidly in those in middle age than in those of advanced age (85+). Overall, combining all ages and both sexes, the age-standardised mortality rate based on all mentions of IHD (excluding myocardial infarction) on the death certificate in 2008–2010 was 54% of that in 1981–1983 (table 3).

**Table 3:** Ischaemic heart disease (excluding AMI) in the Oxford region 1981–1983 and 2008–2010

	Number of deaths		Rates per 100 000		Ratio* of rate† in 2008–2010/ 1981–1983
Age group, years	1981–1983	2008–2010	1981–1983	2008–2010	
Men					
35–39	48	27	18.0	8.3	0.46
40–44	72	51	33.3	14.6	0.44
45–49	169	149	84.4	45.9	0.54
50–54	290	180	152.2	64.2	0.42
55–59	561	286	309.3	114.5	0.37
60–64	767	449	477.9	180.7	0.38
65–69	1082	550	825.3	308	0.37
70–74	1243	754	1143.5	522.2	0.46
75–79	1182	1083	1686.2	980.1	0.58
80–84	818	1294	2456.5	1725.3	0.70
85+	628	1829	3876.5	3254.4	0.84
35 and over	6860	6652	453.9	245.2	0.54
Women					
35–39	6	3	2.3	0.9	–
40–44	11	13	5.3	3.7	–
45–49	23	22	12.0	6.7	0.56
50–54	50	36	26.8	12.9	0.48
55–59	137	54	75.0	21.1	0.28
60–64	249	117	145.4	45.8	0.31
65–69	401	159	264.5	84.6	0.32
70–74	671	349	481.7	222.6	0.46
75–79	972	556	894.2	419	0.47
80–84	1023	919	1447	869.4	0.60
85+	1360	2304	2620.4	1954.2	0.75
35 and over	4903	4532	197.2	103.8	0.53
Total	11 763	11 184	307.5	166.7	0.54

Number of mentions on death certificates, age-specific mortality rates per 100 000 population, and the age-standardised death rate in 2008–2010 expressed as a ratio to that in 1981–1983.

*Ratios compare periods covered by different versions of ICD codes and governed by different rules for the selection of underlying cause of death.

†Rates given where deaths with ischaemic heart disease (excluding AMI) failure as a cause in 2008–2010 is ≥15.

AMI, acute myocardial infarction; ICD, International Classification of Disease.

## Discussion

We report a steep decline in mortality rates and modest reductions in absolute number of deaths from heart failure in a large English regional population, over a 30-year period (1981–2010). This decline was observed across all age–sex groups and was confirmed in the English national population over a 16-year period (1995–2010), using all causes of death and not just the underlying cause. Additionally, this decline coincided with dramatic decreases in mortality from AMI and substantial decreases in mortality for (non-AMI) IHD. The declines in cardiovascular death described in this work occurred despite a trend towards reporting of more comorbid conditions and more sensitive diagnostic criteria for myocardial infarction.^13^ The 30-year data did not suggest any plateau or reversal of the declines in more recent years. The smaller decline in numbers than rates reflects a growing and ageing population.

The availability of all-cause mortality data for a regional population of England (Oxford) has allowed us to consider whether part of the decline in mortality from AMI and other IHD was accompanied by a ‘counter-prevailing’ increase in mortality from heart failure. It was not; mortality rates for heart failure fell substantially, more than the rates for IHD (excluding AMI), though not as much as the rates for AMI. The additional information provided by inclusion of mentions of heart failure is particularly useful given that heart failure, when certified, is uncommonly used as the ‘underlying cause of death’. Furthermore, analysis of all certified causes is necessary as there have been artefactual changes in trends seen from heart failure as the underlying cause.^8^ There was an apparent dip in underlying cause mortality rates for heart failure between 1984 and 1992. This occurred because of changes in death certification rules, which were subject to particularly rigorous implementation in England from 1984 to 1992.^14^

Heart failure hospitalisation and mortality rates after admission were found to decline for elderly Medicare beneficiaries from 1998 to 2008.^7^ However, these results may represent a shift towards treatment of heart failure in an outpatient setting, as has previously been observed, rather than a true decrease in heart failure admissions or mortality.^15^ Examinations of trends in heart failure mortality in the Framingham and Olmsted County cohorts concluded that the incidence of heart failure had remained relatively constant, but that mortality from heart failure had decreased.^6^ ^16^ However, these analyses are similarly limited by their examination of a specific community, which may or may not be representative of the population as a whole. Our analysis of mortality data demonstrates that heart failure mortality has declined significantly in England across all age groups, at least in the Oxford population, since 1981. We, therefore, present unique evidence that heart failure mortality has declined over a long period of time, in conjunction with the decline in AMI and IHD mortality. The recent evidence from the all-England data set indicates that, at least in recent years, this decline is not an artefact of a specific community or of a specific patient population.

On its own, analysis of death certificates is not sufficient to ascertain whether the burden of heart failure to patients and healthcare providers has decreased. It is possible that, despite the observed decline in rates and absolute number of heart failure deaths, the prevalence of heart failure has increased due to individuals living for longer periods of time with heart failure and other comorbidities (as a result of improved case-fatality rates). However, when taken in conjunction with our analysis and other reports showing dramatic declines in AMI and IHD in the UK,^17^ it seems unlikely that the decrease in heart failure mortality observed here has been accompanied by a discordant rise in disability from heart failure. In the Global Burden of Disease study 2010 for the UK, IHD (which incorporated myocardial infarction and the majority of heart failure cases in that report) was one of the only three causes of disability that showed significant decline;^17^ disability-adjusted life years almost halved for IHD from 1990 to 2010, both in absolute terms and when age-adjusted rates were compared. These large, concomitant declines in IHD, AMI and heart failure mortality may reflect declines in shared risk factors. Indeed, much of the decline in IHD mortality in the UK from 1981 to 2003 can be explained by declines in the rates of smoking, serum total cholesterol levels and blood pressure.^18^

The generalisability of these findings to other countries may be limited. The global increase in cardiovascular disease over the next 20 years will be concentrated in low-income and middle-income countries^19^ where analysis of mortality trends for heart failure is particularly difficult. However, our results indicate that trends observed in AMI and IHD are likely to be paralleled by similar trends in heart failure. This suggests that the burden of heart failure within low-income and middle-income countries may actually increase substantially over the next 20 years.^20^ Even in the UK, despite the observed improvements in mortality for cardiac disease, mortality for IHD is still higher than in many European countries and Australia. Recent findings of the global burden of disease study 2010 show that the UK ranked 17/19 against comparator nations on mortality for IHD in 1990, improving only to 14/19 in 2010,^17^ and control of hypertension as the major risk factors for IHD and HF is not optimal.^21^

In conclusion, the availability of all-cause mortality data for a subset of the England population over three decades, together with national mortality data, has allowed us to present the most complete analysis possible of trends in mortality rates for AMI, other IHD and heart failure in a large defined population in England. These results indicate large and sustained reductions in age-standardised and sex-standardised death rates as well as absolute number of deaths attributed to heart failure, AMI and other manifestations of IHD, with no evidence of a nadir during recent years. The exact reasons behind such dramatic long-term declines are beyond the scope of this study, but are likely to be due to a combination of environmental changes, public health measures and improvements in clinical care.

**What is already known on this subject**

- There have been large declines in acute myocardial infarction mortality and ischaemic heart disease mortality in the UK.
- It is unclear if these declines are associated with a ‘counter-prevailing’ increase in heart failure mortality.

**What this study adds**

- Declines in myocardial infarction and ischaemic heart disease mortality have been paralleled by declines in heart failure mortality.
- The environmental changes, public health measures and improvements in clinical care that have caused the described decline in heart failure mortality require further investigation.
